# Affective Impact on Informal Caregivers over 70 Years of Age: A Qualitative Study

**DOI:** 10.3390/healthcare12030329

**Published:** 2024-01-27

**Authors:** Raimunda Montejano-Lozoya, María del Mar Alcañiz-Garrán, Juan Diego Ramos-Pichardo, Miriam Sánchez-Alcón, Sofía García-Sanjuan, Ángela Sanjuán-Quiles

**Affiliations:** 1 La Fe Nursing School, University of Valencia, 46010 Valencia, Spain; montejano_rai@gva.es (R.M.-L.); garran_mar@gva.es (M.d.M.A.-G.); 2 Department of Nursing, University of Huelva, 21071 Huelva, Spain; miriam.sanchez@denf.uhu.es; 3 Department of Nursing, University of Alicante, 03690 San Vicente del Raspeig, Spain; sofia.garcia@ua.es (S.G.-S.); angela.sanjuan@ua.es (Á.S.-Q.)

**Keywords:** informal caregiving, old age, affective, qualitative research, ageing

## Abstract

Given today’s rapidly ageing society, family members providing informal care to dependent older adults face ever-increasing challenges. The aim of this study was to describe the affective impact on older adults over 70 years of age caring for a dependent older person at home. A qualitative study was designed from a phenomenological perspective. Thirteen in-depth interviews were conducted with caregivers aged 70 or older. A content analysis of the interviews was carried out in five stages. Three themes were identified: “Emotions”, “Feelings”, and “Looking to the future”. Caregivers express negative emotions (sadness, anger, and fear) and feelings of social and emotional isolation, and they feel abandoned by health professionals, family, and friends. In conclusion, prolonged caregiving by older adults has a negative affective impact and creates uncertainty about the future. There is a need to devise social and healthcare policies and actions, creating social support networks to improve their health and emotional wellbeing.

## 1. Introduction

Population ageing is a major demographic challenge for today’s society, entailing considerable costs in terms of the provision of appropriate health and care services, with ageing being closely linked to co-morbidity and dependency [1,2]. Patterns of care appear to be determined mainly by culture, demographics, and household structure [3]. In Spain, familial care in the home is prevalent from generation to generation, with women being the main caregivers, in keeping with a familialist model [2]. Staying in familiar surroundings is often important for older people, so it is increasingly common for them to live alone at home or with their partner. This is becoming more and more common in Spain in particular [3]. Home care is therefore a major demographic challenge, not only because of the associated healthcare costs, but also because of the need to deliver adequate care across all areas of health (physical, psychological, and social) [1,4].

Some older people living at home are supported by a care network providing practical, informative, and emotional support and care. The people in these networks may be formal or informal caregivers. Informal care is the unpaid care of individuals with varying degrees of dependency, provided either by family members or others with no link or obligation to the cared-for person [1]. The number of informal caregivers currently vary from country to country. Across Europe, prevalence rates of informal caregiving range from 20% to 44%, with rates of 4% to 11% for intensive caregivers (more than 11 h per week). In Spain, an estimated 29.9% are informal caregivers, with 9.9% being intensive caregivers [5]. Research by Sundström et al. [3] estimates that older people provide between 22% and 33% of all care hours in Spain, figures that are on the rise as the population of older people in need of care grows.

Given the change in traditional family structures, it is increasingly common in Spain to find homes in which care tasks do not fall on professionals or young family members, but rather older people who care for other dependent older people, who tend to be their spouses or close relatives [6,7].

Faced with this situation, a political strategy was developed in 2007 in order to organise the care of long-term dependent people, creating the Law for the Promotion of Personal Autonomy and Care for People in a Situation of Dependency. However, this system has not covered the desired needs due to the lack of resources, cuts due to an economic crisis, long waiting lists, and the unequal application of the law in the different regions. In this way, the expected aid has not been provided and dependent people continue to be cared for in their homes [8].

Caregiver burden has been linked to reduced life satisfaction [9], lower quality of life [10], and poorer self-reported health status [11]. Brown and Cohen found that caregiving leads to poorer mental health, with significant results, although the data collected were self-reported and an adjusted β coefficient of 1.26 was obtained [12].

Caregiving can also be stressful for caregivers, leading to psychological distress and physiological responses that make illness more likely [13]. Some studies have found that informal caregiving is associated with higher rates of depression and anxiety in caregivers [14,15]. The level of dependency and the intensity of care are also factors adversely affecting the health of caregivers [1,16], having detrimental effects on their physical, mental, and psychological state [4]. These factors are modulated by gender differences, advancing age, low educational attainment, existing poor health, and lack of social support [17]. Consequently, older caregivers may be more vulnerable to the risk of mental health problems due to their older age and caregiving role [18].

Considering the psychological distress of caregivers, it is pertinent to address this problem through interventions, not only when the factors manifest themselves, but also through preventive work before adverse effects occur [19].

Interventions based on the needs of the caregiver and the family are very important to mitigate the burden of dependent people so they are able to cope and strengthen their resilience [20,21].

For this reason, it is essential that caregivers work together with the family and with mental health professionals such as psychiatric nurses, who know how important this situation is for caregivers and understand the experience they are going through. This vision is fundamental to focus on the development of tasks and improve the quality of life of the caregiver through the planning, execution, and evaluation of interventions [22].

Research into caregiving tends to focus primarily on exploring how caregiving tasks are performed while disregarding the emotional and relational aspects of providing care [23]. However, the human world is made up of and lived through emotions [24].

The affective dimension also affects the emotional stability of caregivers. On the one hand, caregivers can experience satisfaction and emotional gratification when they feel that they are doing a positive action in the lives of care recipients. These feelings can strengthen your commitment and motivation in the caregiving role. On the contrary, the affective dimension also addresses the emotional challenges faced by caregivers, such as the inclusion of feelings of sadness, frustration, or emotional exhaustion, especially when faced with difficult or demanding situations in care [25].

Throughout life, the affective dimension of people is built through the experiences they live through [26]. These experiences cause emotions and feelings that, according to some authors, connect our mental and physical processes [27].

Thus, “affective impact” could be defined as the set of changes in behaviour, emotions, or thoughts that occur, or are intensified, by the experience of a situation that involves a loss, damage, or intense threat, which can be significant or prolonged. It is a sensor that makes us perceive reality, since something we experience affects us and usually appears through emotions and feelings [28].

Caregiving and being cared for can encompass a variety of emotions including love, empathy, gratitude, compassion, hatred, resentment, bitterness, and regret [29]. Emotions are internalised and subjective states of mind are shaped by the socio-cultural and spatial context in which they occur. Emotions are a key aspect of everyday caregiving and of how care relationships are experienced and managed.

The experience of caring for a dependent person not only entails a physical overload, but also an emotional one which is associated with feelings of helplessness, uncertainty about the future, insecurity, feelings of guilt for not being able to care for others, isolation from the outside world, lack of social support, and mood disorders, among others [30,31]. This combination of sensations gives rise to the affective impact on caregivers.

A large number of studies, mostly quantitative, have been conducted on the affective experiences of informal caregivers. Their results indicate that feelings and emotions can have a negative impact on the psychological health of the caregiver, with high levels of perceived stress, lower life satisfaction, and reduced quality of life, which can lead to problems of depression or anxiety [32,33,34,35,36,37,38]. Moreover, these effects produce an aggravation associated with the physical and cognitive ageing of caregivers over time, accentuated when they are older caregivers [39].

Research on caregivers tends to focus on exploring how caregiving tasks are carried out, without taking into account the emotional and relational aspects of the caregiving process. Qualitative research allows us to study people’s interpretation of reality from their perspective and helps us to understand and relate to their situation [40]. For caregivers, it is the most suitable procedure for investigating how they feel, what their expectations are, their wishes, feelings, concerns, etc.—everything involved in the caregiving experience.

However, most of the literature includes all informal caregivers, without focusing on older caregivers over the age of 70 who face the responsibilities of managing the needs of a family member in addition to their own illnesses and difficulties that come with their age. Since the affective health of this population is understudied, it is essential to understand their experiences through qualitative methods to improve their emotional and physical wellbeing and to enhance the quality of care they provide. Knowing the affective impact on elderly caregivers is very important in order to provide them with adequate support through interventions in the social and health system, taking into account their socio-affective experiences.

Within this conceptual framework and given the scarcity of studies that address the affective states of elderly caregivers, the objective of this study was to describe the affective impact on the elderly over 70 who care for a dependent elderly person at home.

## 2. Materials and Methods

### 2.1. Design

In order to gain an in-depth understanding of how older caregivers experience the emotional aspects of caring for a family member, we conducted a qualitative study from a phenomenological perspective. In-depth interviews were carried out as they are a suitable method for exploring the participants’ situation from their own point of view [40,41].

Taking into account the objective of the study, an interview script was developed that, without being rigid, would allow us to explore the issues of interest (see Supplementary File). Data collection took place between January and June 2018 in Valencia (Spain).

The interviews focused mainly on the caregiver’s daily life experiences. All of the questions addressed fundamental issues in their life: their expectations, their feelings, their satisfaction, the coexistence and relationship with the care recipient, their social relations, their hobbies, their worries, the length of time they have been a carer for, their difficulties, their motivations, and the purpose of the care provided.

### 2.2. Participants

For the study, 13 caregivers over 70 years of age underwent in-depth interviews. The size of the sample was determined by the saturation of data, reached after the aforementioned 13 interviews.

Respondents were purposively selected from primary care units in the province of Valencia. The community nurse, as the person familiar with the people in the family units, received information about the study and briefed the potential participants. The inclusion criteria were as follows: people over 70 years of age caring for dependent people in need of care and attention, living in the same household as the care recipient, and having the communicative capacity necessary to be interviewed.

Care recipients lacked intellectual, physical, or sensory autonomy caused by age or chronic illness (cardiovascular diseases, respiratory diseases, osteoporosis, osteoarthritis, diabetes, neurodegenerative diseases, mental disorders, and palliative diseases). They were dependent people who needed help and care in carrying out the basic actions of daily life, such as personal care, mobility, housework, and decision making.

After receiving confirmation of participation, those who agreed to take part were contacted by telephone and an interview appointment was arranged. The interviews were conducted in the participants’ homes, on a day and at a time that was convenient for them. They lasted approximately 60 min and were recorded with an audio device, following the signing of an informed consent form. The data collection and content analysis led to the emergence of different categories. Interviews were discontinued when data saturation was reached [42].

### 2.3. Instruments

#### 2.3.1. Zarit Caregiver Burden Assessment

This is a self-administered questionnaire that assesses the burden of caregivers of dependent people [43]. It consists of 22 items with 5 response options on a Likert scale (1 = never to 5 = almost always). The summary score ranges from 22 to 110 points, where the higher the score, the greater the caregiver’s burden. For this study, the Spanish version was used, with a Cronbach’s alpha of 0.92 [44].

#### 2.3.2. Caregiver Strain Index

This is a 13-item self-administered questionnaire with a dichotomous response (true or false) that assesses the level of perceived overexertion and fatigue in the performance of the role of caregivers for dependent people [45]. The total score has a range between 0 and 13 points. Scores equal to or greater than 7 indicate a high level of overexertion and fatigue. For this study, the Spanish version was used, adapted from the original by López and Moral [46], with a Cronbach’s alpha of 0.81.

### 2.4. Data Analysis

All interviews were transcribed verbatim by the lead researcher. The texts were analysed using the five-step process described by Giorgi [40,41]: identification of meaning units, eidetic reduction, phenomenological reduction, the essential structure of experience, and a definitive and unique description of the phenomenon under study.

Two independent researchers (MMAG and RML) conducted a thorough preliminary reading of the interviews to obtain a first impression of them in relation to the stated objective. They then reread the transcripts in more detail, highlighting fragments of text (words, phrases, or sections) that referred to the affective impact that caregiving had on the respondents. The text fragments identified by both researchers were treated as units of meaning, while discrepancies were resolved in consensus meetings with a third researcher who was not involved in the previous phase (JDRP).

The units of meaning were then condensed, abstracted, and labelled with codes. The codes were compared on the basis of similarities and differences, and following discussion and review by the authors, agreement was reached on the final grouping of the codes into categories based on the manifest content of the interviews. Following this inductive phase and using an interpretative process in group meetings of the research team, the categories were grouped into sub-themes and these were grouped into themes, constituting the latent content or underlying meaning. Atlas-ti v8 software was used as a tool to support the whole process.

### 2.5. Trustworthiness

Several procedures were carried out to ensure the credibility, reliability, confirmability, and transferability of the information [47]: two researchers independently carried out the identification of meaning units; the authors of the study through consensus meetings carried out the coding and condensation process; a sample of older caregivers from different backgrounds and of different genders was selected for the study; and the results were compared with other knowledge and studies.

### 2.6. Ethical Issues

The participants’ collaboration was voluntary and altruistic. Written informed consent was obtained from each and every participant, in line with the principles set out in the Declaration of Helsinki and the Belmont Report [48]. The project was approved by the Biomedical Research Ethics Committee of La Fe University and Polytechnic Hospital (Registration No. 2012/0525).

## 3. Results

The sample consisted of 13 people over 70 years of age (8 women and 5 men). The relationships were as follows: 10 married caregivers (76.9%), with an average of 46 years of cohabitation, who have been caring for their spouse for an average of 11.6 years; a daughter with 9 years of cohabitation and 5 years of caregiving; a sister-in-law with 3 years of caregiving; and a friend who has been providing care for 15 years. In terms of educational attainment, 69% had a primary education, 15.3% had a secondary education, and 15.3% had no education. Subjective burden was assessed using the Zarit test [43,44] and the Caregiver Strain Index [45,46]. Eight (61.5%) people displayed a high level of overload and ten (76.8%) showed subjective overload (Table 1).

Analysis of the data identified three main themes confirming the affective impact on the caregivers interviewed: “Emotions”, “Feelings”, and “Looking to the future”. Several sub-themes emerged from these, which can be seen in Table 2. Each of the themes is described below drawing on the participants’ accounts:

### 3.1. Emotions

Emotions of joy, fear, sadness, and anger emerged from the first theme.

The emotional experience with grandchildren when they are young is always pleasant, satisfying, and full of joy. One caregiver expressed his joy when talking about his grandchildren.

*“(…) this afternoon we’re going to pick her up from school (...), she’s a bundle of nerves [laughter, he shows me photos of his grandchildren], she’s six years old now in June and the other one is 14 months old, he’s just started to walk...they’re starting to jump on the beds, and young as he is, I picked him up the other day and threw him on the bed and he laughed...They’re a delight.”*

E11

Respondents frequently expressed fear in relation to various situations such as “being alone”, “having to make decisions”, “the dependent person’s illness getting worse”, “the illness itself”, and “the imminence of one’s own or someone else’s death”.

*“I’m afraid this [points to his head] will stop working.”*

E2

*“You know, [annoyed] I’m not handling it well, I’m dealing with it because I have no choice, there’s no other solution, but I’m afraid, I’m always on my own.”*

E4

Older people were aware of the passage of time, displaying emotional lability and sadness in their accounts.

*“(…) he’s 88 and I’m 86, that’s a long time [there is an emotional silence, he lowers his head and she cries].”*

E13

*“(…) I never do anything major, but lots of little things have happened to me over the years. He remembers more than me [she cries] (...) I get by as best I can. (…).”*

E13

Awareness of their memory loss and physical limitations triggered a sense of rage (anger). In their accounts, it was clear that they downplayed these symptoms as being “age-related” and, from what they said, were resigned to and did not view these symptoms as a risk to their own self-care and the care of the dependent person.

*“(…) I don’t remember things, that’s the worst bit [annoyed], my memory is really bad now.”*

E4

*“I have always made the trip to visit the Virgin and I really enjoyed it (...) These days taking buses is scary and infuriating, I haven’t been there for years, I can’t do it.”*

E3

### 3.2. Feelings

In their responses, participants expressed feeling of helplessness about their current caregiving situation, talking about social and emotional isolation, longing for the past, and even blaming their situation on the cared-for person.

Two caregivers expressed helplessness when dealing with situations of high dependency or the deteriorating health of the people they care for.

*“Tonight [his daughter is leaving on a trip] she’s going away and tomorrow she won’t be here, she’s already told me, of course, so being on my own, what should I do, call SAMU? I have no idea...”*

E5

*“(…) I don’t want to think about it, but, if she ends up bedridden, what do I do? I can’t move her, I’m like this all day [she cries] (...) the thing is, how can I leave her? [Silence] I’ll have her as long as I can, and I don’t want to think about afterwards, I don’t want to think about anything, whatever it is, I don’t want to think about anything because if I do it upsets me.”*

E12

Some caregivers reported that years of caregiving for a highly dependent person who does not react to any stimulus leads to emotional isolation due to having no confidant, no camaraderie, no physical contact (caresses; intimacy), no communication, and nerves.

*“(…) he’s not grateful, if he at least had a kind word to say to me, anything, but now all that’s over, everything…”*

E3

*“I don’t leave the house [he looks at his daughter] in case she falls, she [his wife] can’t be left alone.”*

E7

According to the caregivers’ own accounts of the care received from the primary care team, they report social isolation on the part of both professionals and the family, evidenced by a lack of information on the evolution or outlook of the dependent person’s illness. Caregivers do not feel that they are being heard, and they feel abandoned and frustrated.

*“(…) I shower her, dress her, and try to get her to move, the doctor has told her that the less she sits the better, but they “tell me off” and of course I don’t stop, I live for her [annoyed].”*

E7

*“(…) The doctor is not very keen on giving out prescriptions. What do we do with the mattress, it’s no longer any use, it’s broken, because the compressor makes a lot of noise and the mattress is half empty, something isn’t working properly, it’s been working for four years, 24 h a day, so it’s bound to break down, isn’t it? [He explains and expresses himself vehemently], but then nothing [with resignation]. (…).”*

E5

*“My daughter said to me: “I won’t live to be your age, mum”, do you know what I replied, I would like you to live to see the disappointments that come and go and that they are good ones right? (...) But I wanted something else...When they come to see us, they always rush off, like a doctor’s visit, you could say.”*

E13

*“I don’t go out anywhere, I don’t talk to anyone and I’ve been like this for a long, long time, I can’t talk to anyone, not at all.”*

E8

The caregivers reported that when their daughters needed help in bringing up their grandchildren, they were always available, and now the care is not reciprocated, leading to feelings of wistfulness and reproach.

*“My daughter worked and left the baby girl with me, now “she‘s 22 years old”, I had her all day, they dropped her off in the morning and picked her up in the evening and now what?”*

E9

On the other hand, as they grow up, the relationship seems to become more formal and, consequently, less rewarding. It is perceived as more distant, existing in very different, sometimes incomprehensible worlds, where the once strong reciprocal relationship has largely lost its meaning, with their role within it diminishing as their competence as caregivers declines. They expressed feelings of longing in light of the distance from their grandchildren.

*“The grandchildren? They’ve grown up and I don’t even see them. When my children come, I don’t even ask about them, they’re old enough to ask and worry about their grandparents and they don’t, so to hell with them [she cries and he gets annoyed].”*

E13

A woman describes her grief and longing at not being able to see her brother, blaming her husband for the situation.

*“(…) I have a brother who lives in the village, he’s so lovely [she cries] but now, with this one [her husband], I feel bad crying, we don’t see each other, he also has a wife who is very frail, and in pain.”*

E3

As they spoke, negative feelings emerged, such as reproach, victimisation, and blaming the dependent person for the situation, brought on by ignorance.

*“(…) The doctor gave him two and a half months to live...that was four years ago. Sometimes he says to me “he hasn’t half fooled us”, well yes ......I don’t hold it against him, but that’s the situation.”*

E5

*“He sits all day long... He doesn’t get up at all, he doesn’t move...always in front of the box, always sitting in front of the TV, and that’s why he has poor circulation.”*

E1

*“(…) he’s depressed, my husband doesn’t speak at all, not a word, nothing, and this hurts me a bit, it’s all over [irritated]. I get angry with him, seeing him do nothing, making so little effort, we all think that, he hasn’t made an effort and of course, he’s in a wheelchair, he doesn’t do anything, I think he doesn’t have much willpower.”*

E3

*“She makes me talk a lot, I’ve been through a lot with her, I’ve cried a lot because of her, not because she’s mean, but she gets on my nerves.”*

E12

### 3.3. Looking to the Future

The interviewees think more about the present than about setting future goals. When they talk about the present situation the sub-themes of hope, despair, and uncertainty emerge from their accounts.

*“I have no problems living in the present. I am well thought of, and well looked after. I hardly think about anything anymore. From here on, I could be struck down by something any day and I’ll be right out of here, I don’t think about anything, as a 90 year old, I think that this [his wife’s situation] is going to end soon.”*

E4

A caregiver looks to her future by planning who will care for her when she becomes dependent. Another caregiver spoke about hopes and dreams, imagining an unrealistic future.

*“I would like to have a woman like this (pointing to herself) when I can’t take care of myself.”*

E9

*“If I had a daughter like the one my mother had [she starts crying], I love my mother very much [there is a lot of pain, emotion] and right now she is very, very much on my mind…”*

E10

One caregiver imagines his future without the dependent person he cares for, expressing hopeful plans for the future.

*“I can see my future if she were to go, that is if I don’t go first. At the moment I think I’d go on holiday, I want to be by myself, anywhere, but on my own. And then, I don’t know what I’ll do, I’ll go to the village, I’ll come and go, as the house is sitting there empty, I’ll go away for a few days. I really like cycling, in fact I’ve always done a lot of cycling, so I’d take my bike with me.”*

E5

One caregiver shares negative thoughts in connection with his highly dependent wife’s situation. He is trying to cope, but despair is causing him to have suicidal thoughts.

*“I’m someone who has always picked myself up and that’s it, that’s how it is and that’s the end of it... but this is bad, really bad. It’s very hard, so hard...if you’ve not experienced it, you have no idea……One time I read in the newspaper “an old man killed his wife and then he killed himself, she had Alzheimer’s” and I thought (this was all before it happened to me), this man is not right in the head, all because she’s ill…… And now I get it, that man had had enough, he had no help and he said “ok, what are we doing here, nothing, we both go and that’s that, no more suffering and she is out of here too”, yes I get it, I do, maybe, of all the things I could do, that’s the best... maybe that’s the best thing to do.”*

E5

Based on their negative experiences of caregiving, they talked about the decision they had made about their future and the uncertainty they felt at the thought of their daughters having to go through what they were going through and how they did not want that.

*“If they have to operate on him again, they will probably take him to the long-stay hospital and leave him there until he gets better…”*

E1

*“Sometimes I think, what’s the point of all this? For nothing, from now on, what’s left for me? Really, what’s left for me, to make work for my daughters? From the bottom of my heart, God forbid, because as I know what it is like, I don’t want this for them. My mother spent 15 years with Alzheimer’s, I don’t want this for my daughters.”*

E8

Another caregiver voices aloud uncertainty about his contradictory thoughts. He asks himself whether the care he provides for his wife makes sense and whether it has anything to do with her state of health. He wonders if she would consent to it, if she could decide for herself.

*“(…) we don’t know if we are doing the right thing or the wrong thing, we just don’t know, sometimes I think about it, we never talked about it before, am I doing the right thing in taking care of her or not? What would she prefer, to go or to stay? [Long silence] She has no preference, because she doesn’t think... But she must be taken care of, here she is... well, I see it like this, here she is. [Silence], I think we have to take care of her, until God decides to take her away....We know what’s going on, she’s a sick person who can’t be cured, but in the end, here she is.”*

E5

## 4. Discussion

The aim of this study was to describe the affective impact on adults over 70 years of age caring for a dependent older person at home. The participants’ accounts suggest that day-to-day caregiving is exhausting and places a significant emotional burden on them. What distinguishes this study from the existing literature is its cultural and social context, describing as it does the emotions expressed by people over 70 years of age who provide home care for dependent older people in the family environment, in most cases, their spouses.

The first theme to emerge concerned how day-to-day caregiving affects their emotions. Only one person described the joy of seeing and spending time with their grandchildren. As indicated by some authors, certain protective factors can mitigate the negative effects associated with caregiving and promote better physical and mental health linked to family and emotional ties [4]. In this study, happiness could be a protective factor indicative of reciprocity in family relationships.

However, respondents mainly express negative emotions of fear, sadness, and anger. These emotions could be explained by the caregivers’ advanced age, the provision of long-term and intensive care, and by the objective and subjective overload of the interviewees. It must be borne in mind that daily, round-the-clock care is being provided to dependent older people. The literature on caregiver overload is extensive. It is a problem that has been studied for decades and the physical, psychological, and emotional toll on caregivers is recognised in several studies [1,4,49,50].

The differences observable in this study concerning caregiver burden could be due to certain cultural issues which may influence the way caregivers perceive and respond to care-related challenges. Although the tasks performed by caregivers from different contexts are very similar, such cultural differences also have a major influence on how social support is used, on reciprocity, support between family members, or the way emotions are expressed by both caregivers and the cared-for person [50].

The familialist model of care in which this research is contextualised could explain the results obtained, given that cultural heritage is deeply rooted in the group studied, especially among women. Religion or spirituality, the marriage vows of commitment, and the reciprocity of family care have been passed on by society from generation to generation, with such beliefs being held unconsciously and unquestioningly [2,16,51]. A review study conducted in Europe by Calvó-Perxas et al. [52] describes an association between poor caregiver health and living in a country where caregiving is family-based and involves greater dedication and prolonged care.

Caring for dependent people involves a significant time and energy commitment. Furthermore, heavily involved caregivers are more likely to be emotionally affected, experiencing heightened levels of rage [53], sadness, irritability, or anger [49]. Cook et al. [54] found that older caregivers providing many hours of care per month and assisting with many activities of daily living (ADLs) needs experienced more negative emotions compared to low-intensity caregivers. The same authors also found that mental health problems were more prevalent among women with lower levels of education [54]. Other authors have reported that caregivers who provide care within the home suffer poorer health than those caregivers who provide care outside of the home [53]. Being emotionally close to the cared-for person seems to affect the caregiver’s health because those within the home cannot easily avoid the caregiving situation [55], something that could also account for the results of this study.

With regard to long-term care, the interviewees expressed helplessness, social and family isolation, and longing for the past, and even blamed the care recipient for the situation. Given the tendency for the level of disability to increase as time goes by, more and more hours of caregiving are required, which may cause the caregiver to feel helpless [1,49]. According to Aztaza et al. [49], caregivers take on a significant physical, psychological, and emotional burden. They take responsibility for the life of the dependent person in every way, even making decisions for them. The more time they spend providing care and the longer the illness of the person they care for continues, the harder the situation becomes and the greater the need for help. They see how the cared-for person deteriorates, in some cases feeling guilty about it and developing so-called caregiver syndrome. This is understood as an “inadequate response to chronic emotional stress whose main features are physical and/or psychological exhaustion, a cold and depersonalized attitude in relation to others, and a feeling of inadequacy in the tasks to be performed and therefore guilt” [49]. Some studies have examined loneliness or social isolation among informal caregivers, demonstrating a partial link between informal caregiving and increased loneliness [9,49,56,57]. This is plausible given that prioritising caregiving may reduce the time available for family and friends, resulting in these individuals feeling social and familial isolation.

In this context, social support becomes a necessity for caregivers because it is a source of information, training, and support that can reduce the burden and enhance the process of acquiring caregiving skills. A large social support network and reciprocity within relationships are protective factors for caregivers’ emotional health [4]. Some studies highlight the importance of avoiding loneliness, which may be associated with later morbidity and mortality in caregivers and may also have adverse consequences for care recipients [58].

The emotions stemming from day-to-day, long-term, and intensive care, where caregivers and care recipients are in ever-deteriorating physical, psychological, and social health, lead caregivers to describe an uncertain future with contradictory feelings of hope and despair, leading to feelings of insecurity. Social and emotional isolation, together with the dependency of the cared-for person, fear of losing the loved one, or fear of becoming physically or mentally ill, causes despair and may even lead to suicidal thoughts. Along these lines, some authors describe ambivalence between hope and despair among older adults, optimism about the time they have left to live, and at the same time, despondency about approaching the end of their lives [59]. This feeling of despair coupled with a sense of anguish upon realising that the time they have left to achieve the goals they set for themselves is “relatively short” may be a key factor in triggering depression, because they feel that they will never succeed, that nothing will work out, and that they will not be able to solve their problems [60]. Moreover, exacerbated feelings of despair may lead them to contemplate suicide as the only way out [61].

At the same time, as some authors indicate, older people tend to seek emotional balance, selecting disengagement and re-evaluation strategies as a way of regulating their emotional life in pursuit of balance and harmony [62], which may account for the hope expressed by some of the interviewees.

Informal caregiving is physically and emotionally demanding and is experienced according to the caregiver’s personal, family, and social resources. The demand for care creates ambiguous loss, triggering contradictory feelings and grieving processes in which uncertainty becomes the central focus of care. It is a life experience that forces the caregiver to redefine their identity, their roles, and the bond with the person they care for. This involves an ongoing effort to adapt to and make sense of the challenges of the caregiver’s day-to-day tasks [63].

It is essential to develop policies and social actions that promote recognition of the new role as caregivers that people over 70 are assuming. The current benefits of social services need to be expanded and modified in order to obtain better social support. These services must be significant and cover the needs of older caregivers, coordinating to achieve their best possible quality of life. Expanding the networks of social support groups, having municipal or provincial coordinators accessible and serving as a bridge with the available resources, providing them with online help through counselling chats, and establishing interventions and programmes to provide them with social skills could be measures to achieve these objectives. Unfortunately, these options are currently occupations that are carried out by associations and non-profit organisations [64].

On the other hand, not only are social measures necessary, but health services must also be expanded, promoting interdisciplinary work in which health teams are aware of the psychological and affective support needs of caregivers, especially those older than 70 years [65]. In addition, it is important to develop comprehensive care which guarantees continuity of care through specialised professionals such as psychiatric nurses [22].

It is undoubtedly a vulnerable population for which psychosocial interventions should be planned which can have a long-term follow-up and thus be evaluated in terms of their effectiveness. Psychiatric nurses are the most appropriate managers to carry out this type of intervention. These professionals act comprehensively, provide the necessary social skills, and provide them with the resources that may be available to them in their environment. In addition, they play a fundamental role in empowering caregivers and their families, providing them with useful skills and the necessary knowledge to manage stressful situations [66,67].

### Limitations of this Study

The main limitation of this study relates to the difficulties in generalising the results, inherent in the qualitative methodology used. However, the level of data saturation achieved indicates an adequate number of interviews.

A limitation of this study that could be considered a selection bias was the inclusion of only participants who resided in the same household as the care recipients.

Another limitation is its data collection period, as the information was collected in 2018, and the results of the study may vary when compared to the present day.

## 5. Conclusions

Based on our results, caring for dependent older people has an affective impact primarily involving emotions and feelings that cause distress. Prolonged and intensive caregiving leads to emotional and social isolation, longing for the past, and even blaming the cared-for person for their situation. Older caregivers feel neglected by health professionals and by their family members, causing them to have mixed feelings about an uncertain future. The dependency and/or loss of the person they care for, or the loss of their own physical and mental capacity to continue providing care, generates fear and despair, even leading to suicidal thoughts.

The affective impact could be due to their advanced age, the number of years of cohabitation with the cared-for person, and the long periods of time spent providing care, all of which constitute a risk factor for their health. It is therefore necessary to devise social and healthcare policies and actions, creating social support networks to improve their health and emotional wellbeing.

## Figures and Tables

**Table 1:** Characteristics of participating older caregivers.

Code/Sex	Age (Years)	Relationship to Caregiver	Years of Cohabitation	Years Providing Care	Number of Children	Level of Education	Caregiver Zarit Test ^a^ Caregiver Strain Index ^b^
E1/Woman	85	Spouse	38	2	0	Primary school	Intense overload (93 p) ^a^ High level of overexertion (11 p) ^b^
E2/Man	75	Spouse	42	38	0	Secondary education	No overload (46 p) ^a^ No overexertion (3 p) ^b^
E3/Woman	75	Spouse	42	8	3	Primary school	Intense overload (77 p) ^a^ High level of overexertion (12 p) ^b^
E4/Man	89	Spouse	61	7	2	Primary school	No overload (46 p) ^a^ No overexertion (5 p) ^b^
E5/Man	85	Spouse	50	10	2	Secondary education	Intense overload (69 p) ^a^ High level of overexertion (13 p) ^b^
E6/Woman	71	Daughter	9	5	1	Primary school	Overload (55 p) ^a^ No overexertion (6 p) ^b^
E7/Man	84	Spouse	45	15	2	Primary school	Intense overload (65 p) ^a^ High level of overexertion (10 p) ^b^
E8/Woman	85	Spouse	28	10	2	No education	Intense overload (61 p) ^a^ High level of overexertion (11 p) ^b^
E9/Woman	82	Sister-in-law	15	3	2	Primary school	Intense overload (68 p) ^a^ No overexertion (6 p) ^b^
E10/Woman	80	Spouse	48	11	2	No education	Intense overload (57 p) ^a^ High level of overexertion (12 p) ^b^
E11/Man	84	Spouse	40	5	0	Primary school	No overload (34 p) ^a^ No overexertion (6 p) ^b^
E12/Woman	81	Friend	44	15	0	Primary school	Intense overload (73 p) ^a^ High level of overexertion (10 p) ^b^
E13/Woman	86	Spouse	60	10	2	Primary school	Overload (54 p) ^a^ High level of overexertion (12 p) ^b^

^a^ Used to assess the subjective overload generated by the provision of care. Scoring: <47 points = no overload; 47–55 points = overload; >55 points = intense overload; p = points. ^b^ Used to assess the objective overload generated by the provision of care. Scoring: ≥7 points = high level of overexertion; p = points.

**Table 2:** Themes and sub-themes arising from the interviews.

Common Themes	Sub-Themes
Emotions	1.1. Joy 1.2. Fear 1.3. Sadness 1.4. Anger
2. Feelings	2.1. Social isolation 2.2. Emotional isolation 2.3. Helplessness 2.4. Longing 2.5. Blaming the cared-for person
3. Looking to the future	3.1. Hope 3.2. Despair 3.2. Uncertainty

## Data Availability

Data are contained within the article.

## Supplementary Materials

The following supporting information can be downloaded at: https://www.mdpi.com/article/10.3390/healthcare12030329/s1.
