# Supplementary material for: Affective Impact on Informal Caregivers over 70 Years of Age: A Qualitative Study
Source: Healthcare (Basel). 2024 Jan 27;12(3):329. doi: 10.3390/healthcare12030329 (PMC10855777; doi:10.3390/healthcare12030329)
Supplement: Supplementary file 1 [file healthcare-12-00329-s001.zip › healthcare-2775523-supplementary.pdf]

Guidance script for a descriptive phenomenological interview

Initial question.

Tell me about a normal day taking care of XXXXX? Please tell me or describe your everyday experience related to caring for XXXX.

Issues to explore

- How long have you been living with the dependent elderly person? How has living been? Have your life expectations in general been met?
- What do you feel when you think about the current moment? Has your experience of caring changed the relationship you have with the dependent elderly person or your family?
- Why do you care for the dependent elderly person?
- Are you satisfied with what you do?
- Do you have moments of "breathing" of "tranquillity", can you relax? Do you have social relationships, friendships? Do you share your worries with someone? What do you feel when you think about the future?
- What are the most frequent difficulties or problems you encounter every day, how could you solve or reduce them?
- What feelings does this situation provoke in you?
- What does caring for old age mean to you?
- What motivates you to help the dependent elderly person?
- Do you feel that you are helping another person? What does caring bring you?
